# Effect of Xueniao Capsule on *Escherichia coli*-Induced Acute Pyelonephritis Rats by ^1^H NMR-Based Metabolomic Approach

**DOI:** 10.1155/2019/6723956

**Published:** 2019-09-03

**Authors:** Xuliang Hao, Pan He, Yan Ni, Jing Yao, Yankun Yang, Cong Liu, Lei Chen

**Affiliations:** ^1^Shanxi Institute of Traditional Chinese Medicine, No. 46 Bingzhou West Street, Taiyuan 030012, China; ^2^Shanxi Institute of Medicine and Life Science, No. 61 Pingyang Road, Taiyuan 030006, China

## Abstract

Xueniao capsule, one of the famous traditional Chinese medicine (TCM) formulas, has been proved to be effective for treating acute pyelonephritis (APN) in the clinic. However, the probable mechanisms are still unclear. This study was aimed at investigating the therapeutic effect and action mechanism of Xueniao capsule on acute pyelonephritis rats. Chemical analysis of Xueniao capsule and four different extracts was conducted by HPLC and GC-MS. 21 compounds were identified in the Xueniao capsule, and obvious chemical difference was also revealed among the different extracts by chemical analysis. Metabolomics, combined with bacteriological examination, traditional histopathology, and biochemical parameters, was used to evaluate the effects of Xueniao capsule and four different extracts. After treatment with Xueniao capsule, the bacterial count of urine was decreased and the renal lesions of APN rats were ameliorated by histopathology inspection. Levels of Scr and Ucr, IL-1*α*, IL-1*β*, IL-6, IL-10, CXCL-2, and MCP-1 were decreased significantly, and the reserving effect of Xueniao capsule was superior to the different extracts and norfloxacin. 16 endogenous metabolites related to APN model were revealed, and 12 of them could be reversed by the Xueniao capsule. ^1^H NMR metabolomic results demonstrated that the formula of Xueniao capsule played the best therapeutic role on APN through regulating energy metabolism and alterations of osmotic pressure. The effect of Xueniao capsule on the APN was the synergistic actions of multiple components, which need to be further investigated in future studies.

## 1. Introduction

Acute pyelonephritis (APN) is one of the most common renal diseases. It is an inflammation of the kidney characterized by the sudden onset (within one to two days) of fever and chills, side pain, and frequent, painful urination. Acute pyelonephritis typically results from a bacterial infection, most commonly *Escherichia coli*, which could be usually spread up the urinary tract [1]. Acute pyelonephritis is considered to be the result of ascending infection from the bladder to the kidney [2]. The majority of APN occurs in women, especially in childbearing age. Complications may include pus around the kidney, sepsis, or kidney failure [3]. Once present, it is generally treated with antibiotics, such as ciprofloxacin, levofloxacin, or ceftriaxone. However, with the wide use of antibiotics, its resistance and other side effects are more and more obvious.

Traditional Chinese medicine (TCM) formula, which is used as a holistic and synergistic approach to restore the body's homeostasis, has been validated in a host of clinical experiences. Xueniao capsule (XNC), a famous TCM formula to treat acute pyelonephritis recorded in “Drug Standards of the Ministry of Health of the People's Republic of China,” consists of three herbs: Coicis Semen, Smilacis Chinae Rhizoma, and Trachycarpi Petiolus seeds. The formula has been reported to possess significant activity against acute pyelonephritis and reduce the damage of renal interstitium in the clinic [4]. However, the probable mechanisms are still unclear.

Metabolomics is one of the systematic biology techniques used for complex disease process and has been applied in the fields of disease diagnosis, biomarker screening, pharmaceutical discovery, and toxicity evaluation [5, 6]. It is consistent with the holistic and synergistic effects of TCM. Metabolomics employed with advanced analysis instruments, multivariate data analysis, and bioinformatics tools was usually used to study the effect and mechanisms of TCM in recent years. NMR is widely used in metabonomic analysis because it shows advantages of high resolution, nonselectivity, simpleness in sample preparation, and rich structural information. Recently, NMR-based metabolomics has been used in a variety of TCM products, such as Baoyuan decoction [7], *Gastrodia elata Blume* [8], and *Ipomoea aquatica* [9]. In the present study, the ^1^H NMR metabolomic approach, combined with bacteriological examination, traditional histopathology, and biochemical parameters, was used to investigate the therapeutic effect of XNC.

## 2. Materials and Methods

### 2.1. Solvents and Chemicals

Xueniao capsules (XNC) were manufactured from Shanxi Zhendong Kaiyuan Pharmaceutical Co. Ltd. (approval number: Z20093146; batch number: 20150504; 0.34 g per capsule). Norfloxacin capsules were bought from Shanxi Taiyuan Pharmaceutical Co. Ltd. (approval number: H14020362; batch number: 150401). D_2_O was purchased from Sigma-Aldrich, and sodium 3-trimethlysilyl [2,2,3,3-*d*_4_] propionate (TSP) was bought from Andover, MA. Protocatechuic acid, catechin, chlorogenic acid, caffeic acid, polydatin, astilbin, engeletin, resveratrol, and quercetin were obtained from the National Institutes for Food and Drug Control (Beijing, China). Other reagents were of analytical grade.

### 2.2. Preparation of XNC Extracts for Chemical Analysis and Animal Experiment

The contents of 150 capsules were extracted thrice under refluxing with petroleum ether (1 : 10, 1 : 8, 1 : 6, w/v) successively each one hour and filtrated. Then the residues were extracted in ethyl acetate, *n*-butanol, and water separately in the same way. The filtrates were condensed below 60°C under vacuum and dried in order to extract petroleum ether (PE) extract, ethyl acetate (EA) extract, and *n*-butanol (BU) extract as well as water extract (W). The yield rates were 7.3%, 1.18%, 13.33%, and 27.36%, respectively. All extracts were stored at −20°C before use.

### 2.3. Chemical Analysis of XNC Extracts by HPLC and GC-MS Fingerprints

2.0 g of XNC was weighed and extracted with 50 mL methanol by refluxing for 60 min. Then, the filtrate was evaporated to dryness, and the residue was dissolved in 50% acetonitrile to 2 mL. The supernatant was filtered through a 0.22 *μ*m PTFE syringe filter (WondaDisc NY, SHIMADZU-GL, Japan) and transferred to an autosampler vial for HPLC-UV analysis. Chromatographic analysis was performed on the UltiMate 3000 high-performance liquid chromatography. Chromatographic separation was carried out at 25°C on a Waters XBridge C_18_ (4.6 mm × 250 mm, 5 *μ*m) column. The mobile phase consisted of acetonitrile (A) and 0.1% phosphoric acid solution (B), and the gradient elution for analysis was set as follows: 0–30 min, 5% A; 30–45 min, 5–13% A; 45–75 min, 13–20% A; 75–85 min, 20–25% A; and 85–100 min, 25–35% A. The flow rate was kept at 1 mL/min, and the injection volume was 10 *μ*L. The wavelength was set at 280 nm. The four extracts, PE, EA, BU, and W, were also weighed proper amounts (PE: 0.15 g; EA: 0.024 g; BU: 0.27 g; W: 0.55 g). PE extracts were dissolved in methanol-chloroform solution (v/v, 7 : 3). EA and BU extracts were dissolved in methanol, and W extracts were dissolved in 50% acetonitrile aqueous solution. Then the HPLC analysis was conducted using the same chromatographic condition as the XNC.

For the GC-MS analysis, 0.2 g of PE extracts was weighed accurately and extracted with 4 ml sulfate-methanol (v/v, 10 : 90) solution by refluxing for 2 h in 70°C water bath. After cooled, 4 mL of *n*-hexane was added to acquire the *n*-hexane layer. Then, the *n*-hexane layer was dried by anhydrous sodium sulfate (Na_2_SO_4_) for GC-MS analysis, which was performed using 6890GC-5973i MS (Agilent, USA). Chromatographic analysis was carried out on a HP-5MS capillary column (30 m × 250 *μ*m, 0.25 *μ*m film thickness; 5% diphenyl cross-linked 95% dimethylpolysiloxane, Agilent). Helium carrier gas was used at a constant flow rate of 1 mL/min. The injection volume was 1.0 *μ*L, the split ratio was 20 : 1, and the temperature of the injection port was 250°C. Programmed heating condition was set as follows: initial temperature was set at 45°C for 2 min, followed by a ramp to 180°C at 5°C/min for 10 min, and then to 250°C at 10°C/min and maintained for 10 min. The ion source and quadrupole temperatures were set at 230°C and 150°C, respectively. MS detection was implemented with electron ionization (electron energy of 70 eV) and full-scan mode (*m*/*z* 50–550).

### 2.4. Animal and Experimental Design

Male Sprague Dawley rats (180–200 g) were purchased from Beijing HFK Bioscience Co. Ltd. (SCXK (Jing) 2014-0004). Before the experiments, the animals were acclimated to a new environment for one week at room temperature (20–25°C) and constant humidity (40–70%) with a 12 h light/dark cycle. The animals had free access to water and food throughout the study. The procedures in this study were performed in accordance with the National Institute of Health guidelines of the Care and Use of Laboratory Animals and approved by the Ethics Committee of Shanxi Institute of Traditional Chinese Medicine.

The procedures of the APN model rats were carried out according to [10, 11]. In brief, following a 12 h fasting period, animals were anesthetized by intraperitoneal (i.p.) injection of pentobarbital (40 mg/kg) and lay on their back on the operating table. Middle incision was made on the inferior belly and then the right ureter was isolated. The ureter was ligated with 0.17 mm silk suture at 1/3 point. The bladders of rats were exposed and 0.5 ml of a freshly prepared *Escherichia coli* (ATCC25922, Shanghai Luwei Technology Co. Ltd.) solution was injected into their bladders. The control group (NS, *n*=10) was injected with 0.5 ml normal saline and the ureter was not tied. Finally, the abdominal incision was sutured, and the animals were returned to their cages with free access to food and distilled water. After 24 hours, the suture was removed and ureters were reopened for all the rats except for the NS group.

APN rats were divided randomly into seven groups (*n*=10), namely, APN group (APN), APN + norfloxacin group (APN + N), APN + Xueniao group (APN + XNC), APN + PE group, APN + EA group, APN + BU group, and APN + W group. Then, the treated groups were administered with norfloxacin (66.7 mg/kg), XNC (1.275 g/kg), PE extract (0.093 g/kg), EA extract (0.015 g/kg), BU extract (0.170 g/kg), and W extract (0.349 g/kg) by gastric irrigation for ten days, respectively. The doses for the different extracts were calculated by the extraction yields, respectively. The NS group was only administered by oral gavage with 1% (v/v) Tween-80 solution, which was used as the solvent for different extracts and positive drug. Rats were fasted for 12 h before the last administration and anesthetized with 10% chloral hydrate i.p. (0.35 ml/100 g). Blood was collected from aorta abdominalis and centrifuged at 3500 rpm for 20 min to afford serum. Urine was collected from the bladder, and the right kidney was taken out immediately. One half of the right kidney from all groups was fixed in 10% formaldehyde for histopathologic examination, and the other half was rinsed and stored at −80°C for metabolomic study.

### 2.5. Assays for Bacteriological Examination

Bacteria were cultured using 20 *μ*L urine by a plate coating method. A quarter of bacterial colony on the plate was scraped off after 24 h and then dissolved in 25 mL saline. The optical density was measured at 600 nm using TU-1901 double-beam UV-vis spectrophotometer (Pu Xi, Beijing, China).

### 2.6. Histopathologic Evaluation

Formalin-fixed kidneys were processed with paraffin embedding machine (Histocentre III, Shandon Finesse) and subsequently sectioned with paraffin section machine (Shandon Finesse 325, UK) at 3 *μ*m thick. These sections were stained with hematoxylin-eosin (H&E) and then assessed by a fluorescence microscopy imaging system (BX53, Olympus, Japan). Renal injury of the rat was evaluated and given a score from 0 to 3 (0 = normal, 1 = slight damage, 2 = moderate damage, and 3 = severe damage) as previously described [12]. The injury scores of all groups were analyzed by SPSS 16.0.

### 2.7. Assays for Biochemical Parameters

The levels of Scr, BUN, IL-1*α*, IL-1*β*, IL-6, IL-10, CXCL-2, and MCP-1 in the serum, as well as Ucr in the urine, were analyzed according to the instructions of commercial kits by Synergy H1 full function microplate detection instrument (American BioTek company). Experimental values were expressed as mean ± standard deviation (SD). A value of *p* < 0.05 using one-way ANOVA (SPSS 16.0) was considered to be statistically significant.

### 2.8. ^1^H NMR Metabolomics Analysis of Kidney Tissue Extracts

Kidney tissues (200 mg) were extracted with methanol/water (1 : 1) by a homogenizer (German IKA) with minor adjustment. The homogenate mixture was centrifuged at 13,000 rpm for 15 min at 4°C, and then, the supernatants were dried under a stream of nitrogen gas. The residue was dissolved in 750 *μ*L phosphate buffer (0.2 M NaH_2_PO_4_/Na_2_HPO_4_, pH 7.4), containing 0.01% TSP and 10% D_2_O. After centrifugation (13,000 rpm, 4°C, 15 min), 600 *μ*L of the supernatant was transferred into a 5 mm NMR tube for analysis. ^1^H NMR spectra were acquired on a Bruker 600 MHz AVANCE III NMR spectrometer (Bruker BioSpin, Germany) at 298 K equipped with a Bruker 5 mm double-resonance BBI probe, using the noesypr1d sequence with water suppression. 64 scans were collected into 65,536 data points over a spectral width of 12,345.7 Hz, with a relaxation delay of 1.0 s and an acquisition time of 2.65 s.

All ^1^H NMR spectra were manually phased and baseline-corrected using the MestReNova software (version 8.0.1, Mestrelab Research, Santiago de Compostela, Spain). The spectra of the kidney were referenced to TSP at *δ* 0.00 and the signal integral was computed in 0.01 ppm intervals across the region *δ* 0.60–9.00. The region of *δ* 4.50–5.20 was removed to eliminate the effects of imperfect water saturation, and the remaining spectral segments for each NMR spectrum were normalized to the total sum of the spectral intensity to partially compensate for concentration differences of the samples to analysis. The normalized integral values were mean-centered and subjected to multivariate pattern recognition analysis using the SIMCA-P 13.0 software package (Umetrics, Umeå, Sweden). Principal component analysis (PCA) was initially used to visualize general clustering, trends, and outliers among the observations. Further partial least squares discriminant analysis (PLS-DA) was used to optimize the difference between the groups. The quality of the model was assessed with the total explained variables (*R*^2^*X* values) and the model predictability (*Q*^2^ values), followed by rigorous 200 permutation tests. Orthogonal projection to latent structure with discriminant analysis (OPLS-DA) was conducted with Pareto scaling and *S*-plot was used to identify metabolites significantly contributing to the group separation. SPSS 16.0 software was used to test the significance of differential metabolites by one-way ANOVA. The value of *p* < 0.05 was considered as statistically significant.

## 3. Results

### 3.1. Chemical Analysis of XNC and Different Extracts

**Figure 1:**
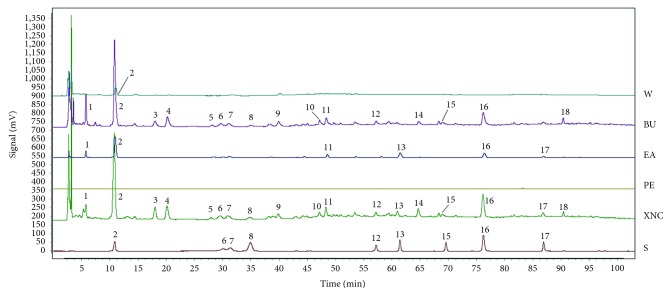
HPLC fingerprints of Xueniao capsule and the four extracts. S, standard solution; XNC, Xueniao capsule; PE, petroleum ether extract; EA, ethyl acetate extract; BU, *n*-butanol extract; W, water extract; 2, protocatechuic acid; 6, catechin; 7, chlorogenic acid; 8, caffeic acid; 12, polydatin; 13, astilbin; 15, engeletin; 16, resveratrol; 17, quercetin.

**Figure 2:**
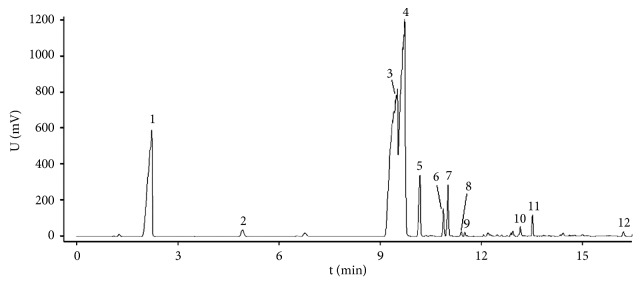
GC-MS fingerprint of petroleum ether extract of XNC. 1, hexadecanoic acid, methyl ester; 2, hexadecanoic acid, ethyl ester; 3, 9, 12-octadecadienoic acid, methyl ester; 4, (E)-9-octadecenoic acid, methyl ester; 5, octadecanoic acid, methyl ester; 6, linoleic acid, ethyl ester; 7, ethyl oleate; 8, 7, 10-octadecadienoic acid, methyl ester; 9, octadecanoic acid, ethyl ester; 10, 11-eicosenoic acid, methyl ester; 11, eicosanoic acid, methyl ester; 12, docosanoic acid, methyl ester.

HPLC fingerprint of XNC is shown in Figure 1. Protocatechuic acid, catechin, chlorogenic acid, caffeic acid, polydatin, astilbin, engeletin, resveratrol, and quercetin were identified by comparison with the reference substances. Then, the PE, EA, BU, and W extracts of XNC were also analyzed using the same HPLC condition. Peak 1, protocatechuic acid (peak 2), peak 11, astilbin (peak 13), resveratrol (peak 16), and quercetin (peak 17) were detected in the EA extracts. Most of the peaks present in the Xueniao capsule were detected in the BU extracts, including protocatechuic acid (peak 2), catechin (peak 6), chlorogenic acid (peak 7), caffeic acid (peak 8), polydatin (peak 12), astilbin (peak 13), engeletin (peak 15), resveratrol (peak 16), quercetin (peak 17), and other unknown peaks. For the W extracts, only peak 1 and protocatechuic acid (peak 2) were detected. As no compounds were identified in the PE extracts, GC-MS was further applied, and 12 fatty acids or their esters were tentatively identified based on the existed NIST2008 library via comparing their MS fragmentation patterns (Figure 2).

### 3.2. Bacteriological Examination

Acute pyelonephritis (APN) was induced by *Escherichia coli* and it is important to measure the bacterial count of urine.  Table 1 shows that the bacterial density of rat urine from the APN group was significantly increased as compared with the NS group. For the drug-treated groups, the bacterial count of urine was decreased, and XNC showed the best effect.

### 3.3. Histopathology

**Figure 3:**
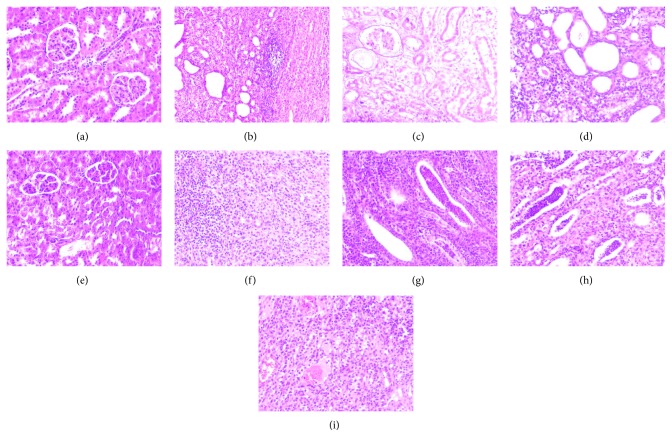
Effect of XNC on histopathology of kidney in rats with acute pyelonephritis (HE × 200; (a) NS; (b, c), APN; (d) APN + N; (e) APN + XNC; (f) APN + PE; (g) APN + EA; (h) APN + BU; (i) APN + W).

The representative images of the histological examination of H&E-stained sections of right kidneys from NS, APN, APN + N, APN + XNC, APN + PE, APN + EA, APN + BU, and APN + W groups are shown in Figure 3. More severe oedema was observed in APN rats, and the main findings of kidneys were multifocal interstitial infiltration with inflammatory cells, mainly neutrophils and lymphocytes, and some dilated tubules with suppurative exudates, whereas no histological abnormalities were observed in the NS group. The lesions in the kidney parenchyma protruded slightly from the surface and a degree of oedema was observed in the APN + N and APN + XNC groups, histologically comparable to the NS group. The renal lesion scores are shown in [Supplementary-material supplementary-material-1]. After treatment with XNC and different extracts, the lesion scores were decreased, as compared with the APN group. Thus, XNC could ameliorate the renal lesions of APN rats, and the effect of XNC was superior to the different extracts.

### 3.4. Changes in Biochemical Parameters

**Figure 4:**
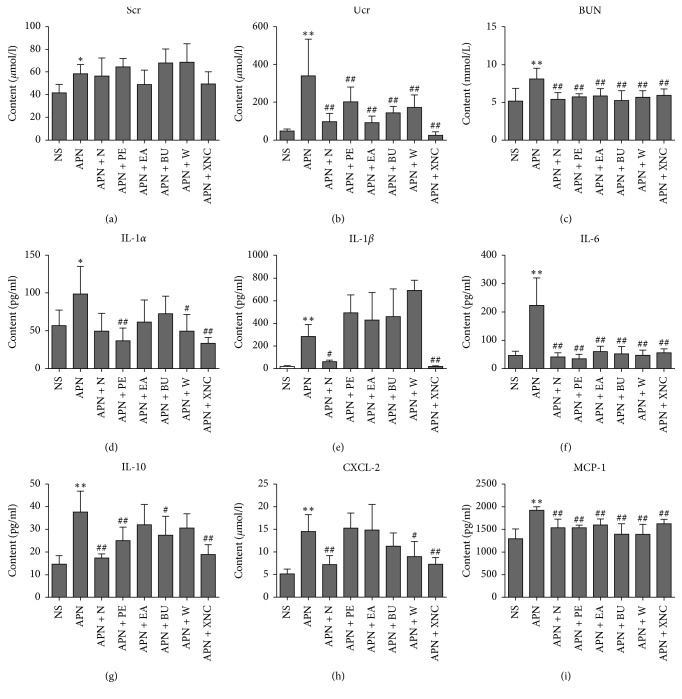
Biochemical parameters of eight groups (*n*=10, $\bar{x}\pm s$) (compared with the NS group, ^*∗*^*p* < 0.05, ^*∗∗*^*p* < 0.01; compared with the APN group, ^#^*p* < 0.05, ^##^*p* < 0.01).

In order to reveal the degree of renal dysfunction, the Scr, Ucr, and BUN [13] were determined and the results are shown in Figure 4. Compared with the NS group, Scr, Ucr, and BUN in the APN group were increased markedly. After the drug treatment, the levels of Ucr and BUN were decreased in all the drug-treated groups, and XNC showed the best reversing effect for the Ucr. For the Scr, XNC also exhibited the best decreasing trend, while the effect of all drug-treated groups was equivalent for the BUN.

Lipopolysaccharide (LPS) of *Escherichia coli* could stimulate NF-*κ*B signaling pathway, which could induce the local secretion of inflammatory (e.g., IL-1*α*, IL-1*β*, IL-6, and IL-10) [14] and proinflammatory (e.g., MCP-1) cytokines and the release of CXCL-1 and CXCL-2 [15]. In this study, the concentrations of IL-1*α*, IL-1*β*, IL-6, IL-10, CXCL-2, and MCP-1 in the serum were measured (Figure 4). The results indicated that levels of these cytokines in the APN rats were significantly elevated compared to the NS group. After the norfloxacin and XNC administration, their levels could be declined significantly. For IL-6 and MCP-1, all the drug-treated groups could prevent their elevation, and there was no difference among the drug-treated groups, while for IL-1*α*, IL-1*β*, IL-10, and CXCL-2, the XNC showed the best reversing effect among all the treated groups. The results suggested that the effect of XNC was superior to the different extracts and norfloxacin.

### 3.5. Metabolomic Changes of Kidney Treated by Xueniao Capsules

Representative ^1^H NMR spectra of kidney samples from eight groups are shown in [Supplementary-material supplementary-material-1]. By analyzing the chemical shifts, peak shapes, coupling constants, and combining with the NMR data reported in the literatures [16, 17], as well as the Human Metabolome Database (HMDB, http://www.hmdb.ca/) and the Biological Magnetic Resonance Data Bank (BMRB, http://www.bmrb.wisc.edu/), a total of 37 endogenous metabolites were tentatively identified, which are listed in [Supplementary-material supplementary-material-1]. The detected metabolites mainly included amino acids, organic acids, and nucleotides.

**Figure 5:**
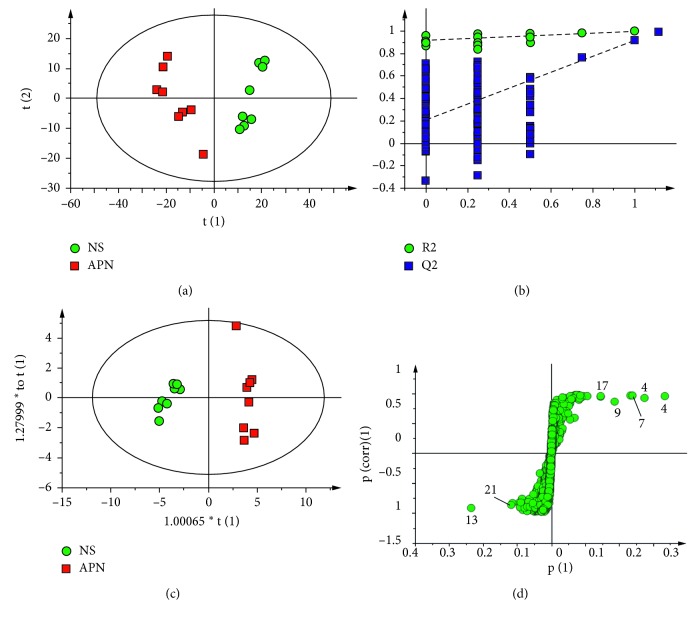
PCA score plot (a), PLS-DA model validation (b), OPLS-DA score plot (c), and *S*-plot (d) of the NS group and APN group.

In order to explore more detailed metabolic differences among the groups, multivariate statistical analysis was applied in this study. PCA score plot was initially used for examining the intrinsic variation of input coordinates, and a clear separation between the NS and APN groups (Figure 5(a), PC1: 0.389; PC2: 0.126) was observed, suggesting that the metabolic disturbance had occurred in the APN rats. The partial least squares discriminant analysis (PLS-DA) model was validated using the response of the permutation test through 200 permutations, in which all *R*^2^ and *Q*^2^ values were lower than original ones. The PLS-DA model parameters (*R*_2_*X*=0.723, *R*_2_*Y*=0.974, *Q*^2^=0.916) indicated excellent predictive power. The corresponding *S*-plots, combined with independent-sample *t*-tests of the relative amounts of metabolites based on the integral regions, revealed that higher levels of lactate, choline, glutamate, fumarate, succinate, glutamine, acetate, myo-inositol, and ethanolamine were observed in the APN model rats as compared with the NS group. Meanwhile, the levels of valine, DMG, TMA, alanine, serine, betaine, and aspartate were decreased in the APN group.

**Figure 6:**
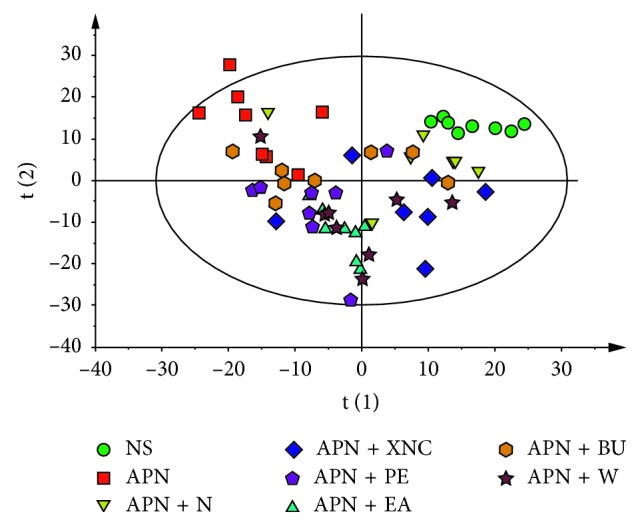
PCA score plot of eight groups derived from ^1^H NMR spectra of kidneys.

To generate an overview of the metabolic response of rats to APN and drug intervention, the PCA of kidney extract profiles of all the groups was conducted (Figure 6). The NS group was completely separated from the APN group, while the APN + N, APN + XNC, APN + PE, APN + EA, APN + BU, and APN + W groups were settled between the NS and APN groups. Furthermore, the APN + N, APN + XNC groups were located closer to the NS group than other groups, indicating that the whole formula of XNC exerted the best therapeutic effect on APN.

The integral levels of APN-related endogenous metabolites of all groups are shown in [Supplementary-material supplementary-material-1]. The results showed that 15 and 12 metabolites were regulated by norfloxacin and XNC, respectively. However, only some of the altered metabolites were reversed by the different extracts, which was in agreement with the bacteriological examination and biochemical parameters. To further quantitatively compare the effect of different treated groups, the EI (efficacy index) was calculated [18]. Ci means the average relative peak area of the metabolite in the NS group. Mi means the average relative peak area of the corresponding metabolite in the APN group. Xi means the average relative peak area of this metabolite in drug-treated groups. The EI value is the total sum of the recovery rates of all recovered metabolites in one group and will be used to quantitatively compare the therapeutic effect on APN. The larger the EI value of one treated group, the better its effect. As shown in Table 2, the EI values of APN + N, APN + XNC, APN + PE, APN + EA, APN + BU, and APN + W were 996.67, 899.74, 337.68, 479.96, 408.61, and 485.55, respectively, indicating that the therapeutic effect of XNC was better than those of its four extracts, and norfloxacin showed the best therapeutic effect on APN according to the degree and number of the metabolites recovered.

## 4. Discussion

APN results from the infection of *Escherichia coli* (Gram-negative bacteria), which could induce the release of inflammatory and proinflammatory cytokines. It was reported that Coicis Semen, containing a large amount of fatty oils, can produce anti-inflammatory effect, which may play a role in adjunctive therapy in Gram-negative bacterial infections [19]. Protocatechuic acid and catechin were effective in preventing cell adhesion and eradicating preformed biofilms of uropathogenic *E. coli* [20]. Engeletin could suppress the expression of TNF-*α*, IL-1*β*, and IL-6 and inhibited NF-*κ*B signaling pathway activation [21], indicating that it exerted anti-inflammatory properties against LPS-stimulated inflammation. Caffeic acid, resveratrol, and quercetin, the natural phenolic compounds, showed obvious anti-inflammatory properties to protect the kidney against acute damage [22–24]. Thus, the effect of XNC was probably related to these components.

**Figure 7:**
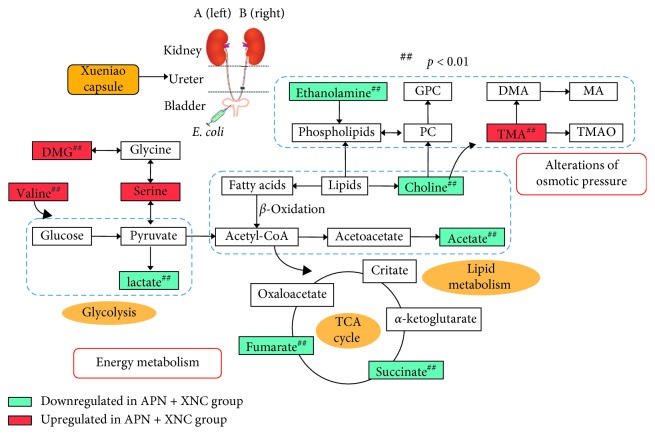
Schematic diagram of the metabolic pathways of the treatment effect of XNC on APN (the rectangle filled with red means the rise whereas the blue means the decline of the metabolites in the APN + XNC group).

The relevant pathways related to the perturbed and regulated endogenous metabolites in the kidney of APN rats (Figure 7) were further discussed based on the KEGG database (http://www.genome.jp/kegg/pathway.html) in detail, which could provide deep insights into the mechanisms of XNC on APN.

### 4.1. Energy Metabolism

One of the most important functions of the kidney is the transfer of sodium ions from the renal tubular fluid to the blood [25]. Energy demand for this process would be provided by mitochondrial aerobic and anaerobic metabolism [17]. Lactate is the end product of glycolysis under anaerobic condition, and its accumulation is a consequence of low metabolism efficiency. In this study, the increased level of lactate in the APN group suggested that glycolysis occurred due to an anaerobic environment. Compared with the APN group, the level of lactate in the kidney was significantly decreased after the administration of XNC, which indicated that XNC could effectively eliminate the accumulation of lactate and promote aerobic metabolizing ability.

Abnormal lipid metabolism in the renal disease has been known for decades [26]. However, the underlying physiological mechanisms of the relationship between lipid levels and progression of renal disease remain poorly understood. Acetate is an end product of fatty acid *β*-oxidation. In this study, the increased level of acetate in the kidney from the APN group denoted a shift in energy metabolism toward fatty acid *β*-oxidation. When the glucose is limited and energy from glycometabolism could not satisfy the demand of the body, fatty acid *β*-oxidation would be enhanced. Downregulation of acetate in the APN + XNC group indicated that XNC could suppress the *β*-oxidation of fatty acids and restore the energy supply.

Succinate and fumarate are the intermediates of the tricarboxylic acid cycle (TCA). The renal mitochondria can use a variety of energy metabolic substrates to produce ATP. The elevated levels of succinate and fumarate in the APN group suggested that an abnormality of the Krebs cycle occurred in the APN rats. After the drug treatment, the decreased fumarate and succinate levels in the kidneys indicated that the activity of TCA cycle could be improved, and the metabolism of succinate and fumarate was accelerated, which further generated sufficient ATP to meet the energy demand.

### 4.2. Alterations of Osmotic Pressure

As one of its normal function, the kidney is known to make use of small-molecule organic osmolytes in maintaining osmotic balance. Choline and carnitine are utilized by intestinal bacteria to produce TMA, and then TMA is oxidized to TMAO by the flavin monooxygenase system [27]. TMAO and myo-inositol play a critical role in protecting renal cells from hyperosmotic stress [28, 29]. Ethanolamine is the second-most-abundant head group for phospholipids substances found in biological membranes (e.g., phosphatidylethanolamine) [30]. The elevated levels of choline, myo-inositol, and ethanolamine in the APN group suggested the kidney structural integrity and functional stabilization of cell membranes were impaired, and osmotic stress was imbalanced in APN rats. After administration with XNC, the levels of choline, myo-inositol, and ethanolamine were decreased, suggesting that XNC could regulate and alleviate the perturbations and maintain the osmotic balance of the kidney.

## 5. Conclusion

XNC is a commonly used formula for the treatment of kidney-related disease in the clinic of TCM, and it was the first study for investigating the therapeutic effect of XNC on APN by the NMR metabolomic approach. The results presented in this study demonstrated that the whole formula of XNC played the best therapeutic role on APN through regulating energy metabolism and alterations of osmotic pressure.

Obvious chemical difference existed among the different extracts, and the results of animal experiments suggested that the effect of XNC was superior to the four corresponding extracts. Therefore, the effect of XNC on the APN was the synergistic actions of multiple components. The polar compounds and nonpolar compounds were both important for the effect of XNC in the APN rats, which reflects the action characteristic of multicomponents and multitargets of TCM. However, the active compounds and their possible synergistic effect in the XNC need to be further investigated in future studies.

## Figures and Tables

**Table 1:** Effect of XNC on urinary bacteria culture of acute pyelonephritis (*n*=10, $\bar{x}\pm s$).

Group	OD_600nm_
NS	0.219 ± 0.065
APN	0.470 ± 0.144^*∗∗*^
APN + N	0.371 ± 0.120
APN + PE	0.210 ± 0.055^##^
APN + EA	0.241 ± 0.045^#^
APN + BU	0.334 ± 0.066
APN + W	0.254 ± 0.038^#^
APN + XNC	0.187 ± 0.053^##^

Compared with the NS group, ^*∗*^*p* < 0.05, ^*∗∗*^*p* < 0.01; compared with the APN group, ^#^*p* < 0.05, ^##^*p* < 0.01.

**Table 2:** Comparison of the integral levels of metabolites in eight groups.

Metabolites	*δ* (ppm)	VIP	APN	Change trend						Recovery rates (%)					
				APN + N	APN + XNC	APN + PE	APN + EA	APN + BU	APN + W	APN + N	APN + XNC	APN + PE	APN + EA	APN + BU	APN + W
Lactate	1.33	9.04	↑^*∗∗*^	↓^##^	↓^##^	↓	↓	↓^##^	↓^##^	67.81^##^	61.72^##^	18.01	18.11	28.89^##^	37.57^##^
Choline	3.21	6.52	↑^*∗∗*^	↓^##^	↓^##^	↓	↓	↓^##^	↓	53.10^##^	47.73^##^	19.99	24.16	36.47^##^	16.76
Glutamate	2.36	2.83	↑^*∗∗*^	↓^#^	↓^##^	↓^#^	↓^##^	↓	↓	26.01^#^	40.02^##^	27.39^#^	45.94^##^	9.32	17.08
Fumarate	6.53	1.01	↑^*∗∗*^	↓^#^	↓^##^	—	↓^#^	—	↓	59.75^#^	69.50^##^	—	80.91^#^	—	42.74
Succinate	2.41	1.92	↑^*∗∗*^	↓^##^	↓^##^	↓^#^	↓	↓^##^	↓	111.21^##^	124.09^##^	71.95^#^	53.85	114.45^##^	53.89
Glutamine	2.13	1.60	↑^*∗∗*^	↓^##^	↓^#^	↓^##^	↓^##^	↓^##^	↓^##^	59.53^##^	28.35^#^	41.81^##^	46.61^##^	52.20^##^	46.14^##^
Acetate	1.93	6.41	↑^*∗∗*^	↓^##^	↓^##^	—	—	—	—	62.80^##^	84.94^##^	—	—	—	—
Myo-inositol	3.53	1.53	↑^*∗∗*^	↓^##^	↓^#^	↓	↓	↓	↓	42.30^##^	35.81^#^	24.93	26.96	30.34	6.22
Ethanolamine	3.15	1.28	↑^*∗*^	—	↓^##^	—	—	—	—	—	138.10^##^	—	—	—	—
Valine	1.05	1.27	↓^*∗∗*^	↑^##^	↑^##^	↑^##^	↑^##^	↑^##^	↑^##^	113.45^##^	117.47^##^	83.79^##^	73.56^##^	90.46^##^	81.38^##^
TMA	2.88	1.05	↓^*∗∗*^	↑^##^	↑^##^	↑	↑^##^	↑	↑^##^	92.11^##^	82.73^##^	42.64	86.78^##^	46.48	89.34^##^
DMG	2.91	1.14	↓^*∗∗*^	↑^##^	↑^##^	—	↑^##^	—	↑^##^	36.92^##^	42.31^##^	—	23.08^##^	—	55^##^
Alanine	1.49	1.14	↓^*∗∗*^	↑^##^	—	↑	—	—	↑	44.35^##^	—	7.17	—	—	9.44
Serine	3.97	1.74	↓^*∗∗*^	↑^##^	↑	—	—	—	—	55.13^##^	26.97	—	—	—	—
Betaine	3.91	3.28	↓^*∗∗*^	↑^#^	—	—	—	—	↑	27.52^#^	—	—	—	—	29.99
Aspartate	2.81	2.33	↓^*∗∗*^	↑^##^	—	—	—	—	—	144.68^##^	—	—	—	—	—
Efficacy index (EI)										996.67	899.74	337.68	479.96	408.61	485.55

Compared with the NS group, ^*∗*^*p* < 0.05, ^*∗∗*^*p* < 0.01; compared with the APN group, ^#^*p* < 0.05, ^##^*p* < 0.01.

## Data Availability

The original spectra of NMR data used to support the findings of this study are provided in the supplementary materials. The HPLC and GC-MS data and biochemical parameters used to support the findings of this study are included within the article.

## Abbreviations

NMR: Nuclear magnetic resonance
APN: Acute pyelonephritis
TCM: Traditional Chinese medicine
XNC: Xueniao capsule
PE: Petroleum ether
EA: Ethyl acetate
BU: *n*-butanol
W: Water
Scr: Serum creatinine
Ucr: Urine creatinine
BUN: Blood urea nitrogen
IL-1*α*: Interleukin-1*α*
IL-1*β*: Interleukin-1*β*
IL-6: Interleukin-6
IL-10: Interleukin-10
CXCL-2: Chemokine (C-X-C motif) ligand 2
MCP-1: Monocyte chemotactic protein 1
TSP: Sodium 3-trimethlysilyl [2,2,3,3-*d*_4_] propionate
PCA: Principal component analysis
PLS-DA: Partial least squares discriminant analysis
OPLS-DA: Orthogonal projections to the latent structures discriminate analysis
EI: Efficacy index
NS: Control group
TMA: Trimethylamine
TMAO: Trimethylamine oxide
DMG: Dimethylglycine
GPC: Glycerophosphorylcholine
PTEE: Polytetrafluoroethylene.

## Supplementary Materials

Supplementary Materials The identification of the major metabolites of NMR spectra from rat's kidney is included within the supplementary information file.
