# Supplementary material for: Effect of Xueniao Capsule on Escherichia coli-Induced Acute Pyelonephritis Rats by 1H NMR-Based Metabolomic Approach
Source: Evid Based Complement Alternat Med. 2019 Sep 3;2019:6723956. doi: 10.1155/2019/6723956 (PMC6745139; doi:10.1155/2019/6723956)
Supplement: Supplementary Materials — The identification of the major metabolites of NMR spectra from rat's kidney is included within the supplementary information file. [file 6723956.f1.doc]

Table S1 The renal injury scores among all groups in histopathology (
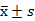
, n=5)

| group | score | group | score |
| --- | --- | --- | --- |
| NS | 0 | APN+PE | 2.40±0.24* |
| APN | 3.00±0.00** | APN+EA | 1.80±0.20* |
| APN+N | 0.40±0.24** | APN+BU | 1.20±0.20* |
| APN+XNC | 0.40±0.24** | APN+W | 1.60±0.24* |

Compared with NS group, **p* < 0.05, ***p* < 0.01; compared with APN group, ^#^*p* < 0.05

Table S2 The identification of the major metabolites of ^1^HNMR spectra from rats kidney

| Keys | Metabolites ^a^ | Moieties | δ ^1^H (multiplicity ^b^ ) |
| --- | --- | --- | --- |
| 1 | isoleucine | δCH_3_, γ'CH_2_ | 0.94(t, J=7.2Hz), 1.01(d, J=7.2Hz) |
| 2 | leucine | δCH_3_,δ'CH_3_ | 0.96(d, J=6.0Hz), 0.97(d, J=6.0Hz) |
| 3 | valine | γCH_3_, γ'CH_3_ | 1.00(d, J=6.9Hz), 1.05(d, J=6.9Hz) |
| 4 | lactate | βCH_3_, αCH | 1.33(d, J=6.6Hz), 4.12(q, J=7.2Hz) |
| 5 | lysine | βCH_2_,δCH_2_,γCH_2_ | 1.91(m), 1.73(m), 1.45(m) |
| 6 | alanine | βCH_3_, αCH | 1.48(d, J=7.2Hz) |
| 7 | acetate | CH_3_ | 1.92(s) |
| 8 | methionine | S-CH_3_, βCH_2_,γCH_2_, αCH | 2.14(s), 2.14(m), 2.64(t, J=7.2Hz), 3.85(m) |
| 9 | glutamate | βCH_2_,γCH_2_ | 2.06(m), 2.35(m) |
| 10 | glutamine | βCH_2_,γCH_2_ | 2.14(m), 2.46(m) |
| 11 | succinate | CH_2_ | 2.41(s) |
| 12 | aspartate | half βCH_2_,half βCH_2_ | 2.82(dd, J=17.4, 3.6Hz), 2.68 (dd, J=17.4, 9.0Hz) |
| 13 | TMA | CH_3_ | 2.88(s) |
| 14 | DMG | N-CH_3_, CH_2_ | 2.90(s), 3.71(s) |
| 15 | creatine | CH_3_, CH_2_ | 3.04(s), 3.94(s) |
| 16 | ethanolamine | CH_2_NH_2_, CH_2_OH | 3.15(t, J=5.4Hz), 3.84(t, J=5.4Hz) |
| 17 | choline | N(CH_3_)_3_ | 3.20(s) |
| 18 | GPC | N(CH_3_)_3_ | 3.23(s) |
| 19 | TMAO | CH_3_ | 3.27(s) |
| 20 | taurine | S-CH_2_, N-CH_2_ | 3.27(t, J=6.6Hz), 3.43(t, J=6.6Hz) |
| 21 | betaine | CH_3_, CH_2_ | 3.27(s), 3.90(s) |
| 22 | *scyll*o-inositol | CH | 3.37(s) |
| 23 | glycine | CH_2_ | 3.56(s) |
| 24 | serine | CH, CH_2_ | 3.85(dd, J=4.2, 6.0Hz), 3.99(dd, J=3.6, 6.0Hz) |
| 25 | *myo*-inositol | 2, 3, 4-CH | 4.07(t, J=3.0Hz), 3.63(t, J=9.6Hz), 3.54(dd, J=10.2, 3.0Hz), |
| 26 | allantoin | CH | 5.40(s) |
| 27 | fumarate | CH=CH | 6.53(s) |
| 28 | L-tyrosine | 3 or 5-CH, 2 or 6-CH | 6.90(d, J=8.4Hz), 7.19(d, J=8.4Hz) |
| 29 | histidine | 2-CH, 4-CH | 7.91(s), 7.10(s) |
| 30 | phenylalanine | 2 or 6-CH, 4-CH, 3 or 5-CH | 7.34(d, J=7.2Hz), 7.39(d, J=7.2Hz), 7.42(m) |
| 31 | uracil | 5-CH, 6-CH | 5.81(d, J=7.8Hz), 7.55(d, J=7.2Hz) |
| 32 | cytidine | 6-CH(ring), 5-CH(ring), 1-C'H(ribose) | 7.84(d, J=7.8Hz), 6.07(d, J=7.8Hz), 5.91(d, J=4.2Hz) |
| 33 | xanthine | 8-CH | 7.91(s) |
| 34 | hypoxanthine | 2-CH, 8-CH | 8.20(s), 8.22(s) |
| 35 | formate | HCOOH | 8.46(s) |
| 36 | adenosine | 2-CH(ring), 8-CH(ring), 1-C'H(ribose), 3-C'H(ribose), 4-C'H(ribose) | 8.35(s), 8.24(s), 6.10(d, J=7.8Hz), 4.45(t, J=6.6Hz), 4. 32(q, J=4.8Hz) |
| 37 | nicotinamide | 2-CH, 6-CH, 4-CH, 5-CH | 8.94(s), 8.71(d, J=7.8Hz), 8.25(m), 7.60(m) |

a: TMA, trimethylamine; TMAO, [trimethylamine](javascript:void(0);) [oxide](javascript:void(0);); DMG, dimethylglycine; GPC, glycerophosphoryl choline

b: s, singlet；d, [doublet](javascript:void(0);)；t, triplet；q, quartet；m, multiplet

Table S3 The integral levels of differential metabolites in eight groups

| metabolites | NS | APN | APN+N | APN+XNC | APN+PE | APN+EA | APN+BU | APN+W |
| --- | --- | --- | --- | --- | --- | --- | --- | --- |
| lactate | 75.43±16.738 | 450.88±88.98** | 196.29±48.08^##^ | 219.16±77.49^##^ | 383.27±119.88 | 382.89±56.13 | 342.42±124.46^##^ | 309.82±93.79^##^ |
| choline | 128.36±18.62 | 313.31±42.71** | 215.10±35.65^##^ | 225.04±33.94^##^ | 276.33±85.69 | 268.62±47.48 | 245.85±67.43^##^ | 282.31±65.03 |
| glutamate | 62.34±7.57 | 97.60±8.69** | 88.95±7.28^#^ | 84.29±15.47^##^ | 88.49±12.87^#^ | 82.32±7.32^##^ | 94.50±4.13 | 91.92±8.88 |
| fumarate | 3.74±1.51 | 8.56±2.19** | 5.68±0.87^#^ | 5.21±1.51^##^ | 14.22±6.05 | 4.66±1.15^#^ | 11.64±5.24 | 6.50±2.81 |
| succinate | 25.17±2.83 | 47.38±2.57** | 22.68±1.93^##^ | 19.82±4.35^##^ | 31.40±12.59^#^ | 35.42±10.52 | 21.96±6.29^##^ | 35.41±13.48 |
| glutamine | 33.48±2.86 | 46.18±2.81** | 38.62±1.26^##^ | 42.58±3.78^#^ | 40.87±3.97^##^ | 40.26±2.30^##^ | 39.55±2.10^##^ | 40.32±2.63^##^ |
| acetate | 66.83±7.24 | 233.16±62.97** | 128.70±34.17^##^ | 91.88±18.82^##^ | 339.13±49.35 | 269.36±40.15 | 258.10±47.34 | 337.40±51.10 |
| myo-inositol | 22.76±2.20 | 37.56±3.44** | 31.30±2.69^##^ | 32.26±4.11^#^ | 33.87±7.83 | 33.57±5.55 | 33.07±4.83 | 36.64±5.65 |
| ethanolamine | 39.61±2.24 | 45.70±7.18* | 44.97±4.24 | 37.29±5.18^##^ | 45.49±10.91 | 49.49±6.42 | 49.59±7.12 | 44.89±6.32 |
| valine | 41.58±3.83 | 32.88±5.25** | 42.75±3.09^##^ | 43.10±3.60^##^ | 40.17±6.90^##^ | 39.28±3.08^##^ | 40.75±4.76^##^ | 39.96±3.03^##^ |
| TMA | 6.53±0.95 | 1.84±0.23** | 6.16±1.79^##^ | 5.72±2.28^##^ | 3.84±1.09 | 5.91±1.22^##^ | 4.02±0.23 | 6.03±1.50^##^ |
| DMG | 6.35±0.97 | 3.75±1.56** | 4.71±2.18^##^ | 4.85±2.17^##^ | 3.63±1.14 | 4.35±0.80^##^ | 2.96±1.19 | 5.18±0.91^##^ |
| alanine | 99.29±6.29 | 68.04±8.71** | 81.90±3.93^##^ | 52.24±14.23 | 70.28±12.80 | 67.56±5.58 | 46.31±11.21 | 70.99±11.13 |
| serine | 38.54±2.12 | 24.30±5.18** | 32.15±5.50^##^ | 28.14±5.67 | 15.70±5.48 | 17.42±2.03 | 21.48±6.44 | 17.54±4.64 |
| betaine | 112.99±17.53 | 59.64±14.14** | 74.32±12.18^#^ | 44.08±4.82 | 42.87±11.22 | 40.59±4.99 | 59.72±15.70 | 75.64±12.34 |
| aspartate | 45.45±2.66 | 21.84±5.74** | 34.16±6.28^##^ | 18.25±4.46 | 16.94±7.88 | 19.42±3.46 | 22.68±8.69 | 18.08±5.31 |

Compared with NS group, **p* < 0.05, ***p* < 0.01; compared with APN group, ^#^*p* < 0.05, ^##^*p* < 0.01


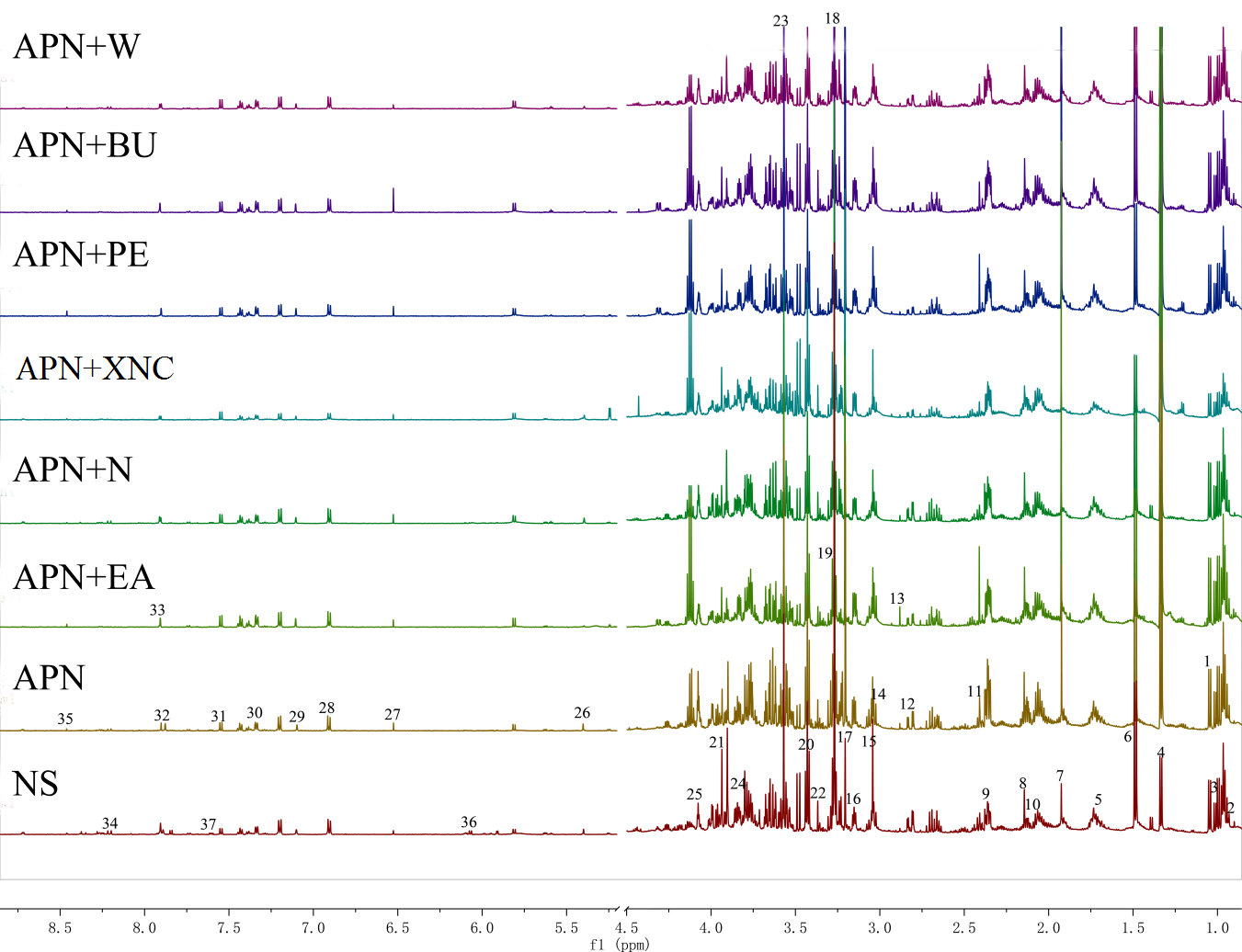


Figure S1 The typical ^1^H NMR spectra of kidney tissues from eight groups
